# Pakistanis living in Oslo have lower serum 1,25-dihydroxyvitamin D levels but higher serum ionized calcium levels compared with ethnic Norwegians. The Oslo Health Study

**DOI:** 10.1186/1472-6823-7-9

**Published:** 2007-10-18

**Authors:** Kristin Holvik, Haakon E Meyer, Anne Johanne Søgaard, Egil Haug, Jan A Falch

**Affiliations:** 1 Institute of General Practice and Community Medicine, University of Oslo, PO Box 1130 Blindern, NO-0318 Oslo, Norway; 2 Division of Epidemiology, Norwegian Institute of Public Health, Oslo, Norway; 3 Center of Endocrinology, Aker University Hospital, and Faculty of Medicine, University of Oslo, Norway

## Abstract

**Background:**

Persons of Pakistani origin living in Oslo have a much higher prevalence of vitamin D deficiency and secondary hyperparathyroidism but similar bone mineral density compared with ethnic Norwegians. Our objective was to investigate whether Pakistani immigrants living in Oslo have an altered vitamin D metabolism by means of compensatory higher serum levels of 1,25-dihydroxyvitamin D (s-1,25(OH)_2_D) compared with ethnic Norwegians; and whether serum levels of ionized calcium (s-Ca^2+^) differ between Pakistanis and Norwegians.

**Methods:**

In a cross-sectional, population-based study venous serum samples were drawn from 94 Pakistani men and 67 Pakistani women aged 30–60 years, and 290 Norwegian men and 270 Norwegian women aged 45–60 years; in total 721 subjects.

**Results:**

Pakistanis had lower s-1,25(OH)_2_D compared with Norwegians (p < 0.001). Age- and gender adjusted mean (95% CI) levels were 93 (86, 99) pmol/l in Pakistanis and 123 (120, 126) pmol/l in Norwegians, p < 0.001. The difference persisted after controlling for body mass index. There was a positive relation between serum 25-hydroxyvitamin D (s-25(OH)D) and s-1,25(OH)_2_D in both groups. S-Ca^2+ ^was higher in Pakistanis; age-adjusted mean (95% CI) levels were 1.28 (1.27, 1.28) mmol/l in Pakistanis and 1.26 (1.26, 1.26) mmol/l in Norwegians, p < 0.001. In both groups, s-Ca^2+ ^was inversely correlated to serum intact parathyroid hormone levels (s-iPTH). For any s-iPTH, s-Ca^2+ ^was higher in Pakistanis, also when controlling for age.

**Conclusion:**

Community-dwelling Pakistanis in Oslo with low vitamin D status and secondary hyperparathyroidism have lower s-1,25(OH)_2_D compared with ethnic Norwegians. However, the Pakistanis have higher s-Ca^2+^. The cause of the higher s-Ca^2+ ^in Pakistanis in spite of their higher iPTH remains unclear.

## Background

Vitamin D deficiency and secondary hyperparathyroidism as defined by low serum 25-hydroxyvitamin D (s-25(OH)D) and elevated serum parathyroid hormone (s-iPTH) are common in immigrants from the Indian subcontinent to Western countries [1,2], including Norway [3]. Some researchers have found lower bone mineral density (BMD) in immigrated Asian Indians and Pakistanis with a low vitamin D status compared with the Caucasian host population [4,5]. In Oslo, however, Pakistani immigrants (30–60 years) do not differ in bone mineral density from ethnic Norwegians [6,7], despite lower serum 25-hydroxyvitamin D levels (s-25(OH)D).

It has been speculated whether vitamin D deficient populations may have a modified vitamin D endocrine system involving a compensatory increase in 1-α-hydroxylase activity and thereby increased serum levels of the active vitamin D hormone (1,25-dihydroxyvitamin D) in response to their secondary hyperparathyroidism. This would maintain their serum calcium levels and probably protect against bone loss, by enhancing intestinal calcium absorption and renal tubular calcium reabsorption.

Some small studies have reported higher BMD [8], and higher [9] or similar serum 1,25-dihydroxyvitamin D (s-1,25(OH)_2_D) [10] in African-Americans compared with Caucasians, despite lower s-25(OH)D in African-Americans. Serum ionized calcium (s-Ca^2+^) was similar in African-Americans and Caucasians [9].

In a small US study (n = 18), vitamin D deficient Asian Indian immigrants also had considerably higher s-1,25(OH)_2_D when compared with Caucasians (n = 27) [1], suggesting a compensatory mechanism in the vitamin D endocrine system. S-Ca^2+ ^was similar in the Asian Indians and the Caucasians.

In earlier studies from Norway, lower s-25(OH)D but similar 1,25(OH)_2_D were observed in pregnant and premenopausal Pakistani women in Oslo compared with ethnic Norwegian women [7,11]. Pakistani and Norwegian women had similar s-Ca^2+ ^[7]. In contrast to these older studies, the Oslo Health Study 2000–2001 is a much larger, population-based study including samples of the general population. It covers a wide age range, and it also includes men. It has thus far been found in the Oslo Health Study that although Pakistanis living in Oslo have a very high prevalence of vitamin D deficiency and secondary hyperparathyroidism compared with ethnic Norwegians [3], the two ethnic groups do not differ substantially in bone turnover [12] or bone mineral density [6]. Based on this knowledge our research questions were: Do persons with Pakistani background living in Oslo have increased s-1,25(OH)_2_D when compared with ethnic Norwegians, in order to compensate for low s-25(OH)D? And, as a consequence of altered vitamin D metabolism, does s-Ca^2+ ^differ between Pakistanis and Norwegians?

## Methods

The data were collected as part of the Oslo Health Study (HUBRO), a cross-sectional population-based multipurpose study conducted in 2000–2001, inviting all individuals in the city of Oslo aged 30, 40, 45, 59, 60, 75 and 76 years. The overall attendance rate was 46% (n = 18,770), varying from 36% in 30 years old to 55% in 59–60 years old individuals. The material and methods, including respondent rates, are described in detail elsewhere [13]. As a substudy of HUBRO, bone mineral density and vitamin D status were measured in the period May 2000 to January 2001. The participants recruited for the substudy were random samples of individuals born in Norway (quoted as Norwegians) aged 45, 60, and 75 years, as well as random samples of individuals born in Pakistan (quoted as Pakistanis) of all age groups in the Oslo Health Study. The participants were invited in a random order throughout the study period regardless of ethnic background. For the current data analysis, we excluded those not having their serum vitamin D metabolites measured, and the oldest age group (75–76 years), as there were only three Pakistanis in this age group.

As previously described [12], of the 1281 Norwegians of age 45–60 who were invited to the substudy, 674 (53%) participated, and 584 (46%) had s-25(OH)D analyzed. Of the 608 Pakistanis of age 30–60 who were invited to the substudy, 237 (39%) participated, and 176 (29%) had s-25(OH)D analyzed. An important reason for these discrepancies was that the data collection for the substudy was discontinued January 15^th^, 2001, and was thus not performed on those who should have met before, but met after January 15^th^. However, background characteristics (smoking, BMI, and age) of those who participated in the substudy did not differ from those who met after this date.

In addition, we excluded two Norwegian women and one Pakistani woman due to primary hyperparathyroidism as defined by s-iPTH ≥ 8.5 pmol/L and serum levels of ionized calcium (s-Ca^2+^) > 1.35 mmol/L, and we excluded one subject due to very low s-iPTH combined with high s-Ca^2+ ^(iPTH < 2.0 pmol/L and Ca^2+ ^> 1.35 mmol/L), which possibly indicate malignancy. In addition, two subjects had unknown PTH status due to missing values, and 33 subjects had unknown s-1,25(OH)_2_D due to missing values. Thus, the final sample for analysis consisted of a total of 94 Pakistani men and 67 Pakistani women of age 30, 40, 45, and 59–60 years, and 290 Norwegian men and 270 Norwegian women of age 45 and 60 years; in total 721 subjects.

### Data collection

All participants underwent a simple physical examination and filled in self-administered questionnaires which included information about various lifestyle factors [14]. A non-fasting blood sample was collected from each participant on the day of attendance. Height and weight were measured in light in-door clothing without shoes.

### Blood sample analysis

The serum samples were first stored at -20°C for up to eight weeks at the screening station, and then kept frozen at -70°C until analyzed in the Hormone Laboratory, Aker University Hospital.

S-25(OH)D and s-iPTH (intact PTH) were measured as previously described [3]. S-1,25(OH)_2_D was measured by competitive radioimmunoassay (DiaSorin, Stillwater, MN, USA). Prior to the 1,25(OH)_2_D determination, serum lipids and interfering vitamin D metabolites were removed by chromatography on a C_18_OH column. Cross reaction with 25(OH)D after chromatography is noted to be 0.002%. The intra- and interassay coefficients of variation (CVs) for the s-1,25(OH)_2_D assay were 7 and 14%, respectively. The limit of detection was 12 pmol/l.

S-Ca^2+ ^was measured using an ion-specific electrode (Ciba Corning Diagnostics, Essex, UK). The interassay CV was 2%. S-Ca^2+ ^levels were adjusted to pH 7.40.

The analyses are not adjusted for time since last meal as additional analyses showed that such adjustment did not influence the levels of any of the metabolites.

### Statistical analysis

Statistical tests were performed using the software SPSS for Windows version 14.0. Ethnic differences in s-1,25(OH)_2_D and s-Ca^2+ ^were assessed by one-way analysis of variance (ANOVA) and linear regression. Associations between metabolites were assessed by linear regression and correlation analysis. We corrected for body mass index (BMI) in order to explore whether a difference in s-1,25(OH)_2_D could be attributable to differences in BMI. Additional data analyses were performed stratified on ethnic background, gender and age.

### Ethics

The study protocol was reviewed by the Regional Committee for Medical Research Ethics and approved by the Norwegian Data Inspectorate. Written informed consent was obtained from the participants.

## Results

### Serum 1,25-dihydroxyvitamin D levels in ethnic groups

Mean (95% CI) s-1,25(OH)_2_D for all participants was 117 (114, 119) pmol/l and ranged from 35 to 258 pmol/l. S-1,25(OH)_2_D was considerably lower in Pakistanis than in Norwegians (table 1). S-1,25(OH)_2_D did not differ between men and women within each ethnic group, and did not differ by age. The mean difference between Pakistani and Norwegian men and women combined was 30 (95% CI 23, 38) pmol/l, p < 0.001. When also controlling for s-25(OH)D and s-iPTH, there was still an ethnic difference in s-1,25(OH)_2_D of 11 (95% CI 2, 20) pmol/l, p = 0.014. There was a positive relation between s-25(OH)D and s-1,25(OH)_2_D (In Norwegians: Beta = 0.4 pmol/l increase in s-1,25(OH)_2_D per 1 nmol/l increase in s-25(OH)D; In Pakistanis: Beta = 0.7 pmol/l increase in s-1,25(OH)_2_D per 1 nmol/l increase in s-25(OH)D. P < 0.001 for both ethnic groups). Correlation coefficients were r = 0.35 (p < 0.001) in Pakistanis and r = 0.26 (p < 0.001) in Norwegians.

**Table 1:** Background characteristics and serum levels of 25-hydroxyvitamin D, intact parathyroid hormone, 1,25-dihydroxyvitamin D, and ionized calcium in Norwegians and Pakistanis

	Men			Women		
	Norwegians n = 290	Pakistanis n = 94	P (ethnicity), ANOVA	Norwegians n = 270	Pakistanis n = 67	P (ethnicity), ANOVA

Age (years) ^a^	56 (55, 57)	43 (42, 45)	< 0.001	55 (54, 56)	40 (38, 42)	< 0.001
BMI, mean (SD) kg/m^2^	26.5 (3.4)	27.2 (3.8)	0.10	25.0 (4.1)	29.4 (4.9)	< 0.001
s-25(OH)D (nmol/l) ^a^	74.4 (71.7, 77.1)	30.0 (24.8, 35.3)	< 0.001	76.2 (73.3, 79.1)	26.1 (19.4, 32.9)	< 0.001
Range	27–207	6–68		19–168	6–70	
s-iPTH (pmol/l) ^a^	5.2 (4.9, 5.4)	6.0 (5.4, 6.5)	0.013	5.0 (4.7, 5.4)	6.9 (6.2, 7.7)	< 0.001
Range	1.3–15.5	1.4–13.5		1.2–15.5	2.4–18.0	
s-1,25(OH)_2_D (pmol/l) ^a^	124 (120, 129)	94 (86, 102)	< 0.001	122 (118, 127)	91 (81, 101)	< 0.001
Range	60–251	43–214		35–225	58–258	
s-Ca^2+ ^(mmol/l)^a^	1.26 (1.26, 1.27)	1.28 (1.27, 1.28)	0.031	1.26 (1.25, 1.26)	1.28 (1.27, 1.29)	< 0.001
Range	1.15–1.36	1.19–1.45		1.15–1.39	1.18–1.36	

^a ^Means and 95% confidence intervals, with age adjustment done separately within the genders.

Pakistanis had higher BMI than Norwegians; unadjusted mean 28.1 (95% CI 27.5, 28.7) kg/m^2 ^in Pakistanis and 25.8 (95% CI 25.4, 26.1) kg/m^2 ^in Norwegians (p < 0.001). The ethnic difference in s-1,25(OH)_2_D persisted after adjustment for BMI, although the adjusted ethnic difference in women was now 3 pmol/l smaller. In a linear regression model, BMI was significantly inversely associated with s-1,25(OH)_2_D in Norwegians (decrease of -0.8 pmol/l per 1 kg/m^2 ^increase in BMI, p = 0.036) but not in Pakistanis. The interaction term for ethnic background and BMI on s-1,25(OH)_2_D was, however, not statistically significant.

### Serum ionized calcium levels in ethnic groups

S-Ca^2+ ^was higher in Pakistanis than in Norwegians (table 1) and the values were similar in men and women. S-Ca^2+ ^did not vary by age except for a positive relation with age in Norwegian women. For all age groups, estimates of s-Ca^2+ ^were higher in Pakistanis.

**Figure 1:**
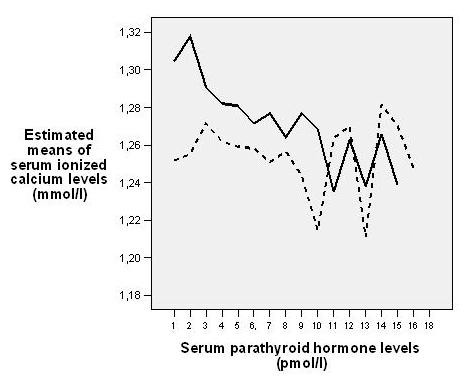
**Age- and gender-adjusted estimated marginal means of s-Ca^2+ ^at different s-iPTH in Pakistanis (n = 161) (—) and Norwegians (n = 560) (- - -)**. Note that there are few observations (n = 46) at serum parathyroid hormone levels above 9 pmol/l.

S-Ca^2+ ^was not correlated to s-25(OH)D or s-1,25(OH)_2_D. However, there was a clear inverse correlation between s-Ca^2+ ^and s-iPTH in both genders in both ethnic groups (overall age-adjusted correlation r = -0.14, p < 0.001), and it was stronger in Pakistanis. Pakistani women, who had the highest prevalence of secondary hyperparathyroidism, had an age-adjusted correlation of r = -0.37 (p = 0.002) between s-Ca^2+ ^and s-iPTH. In the normal range, at any s-iPTH level, Pakistanis had higher s-Ca^2+ ^(figure 1). For s-iPTH above 9 pmol/l, the pattern was unclear due to few observations.

In a multiple linear regression model including ethnic background, age, gender, s-25(OH)D, s-1,25(OH)_2_D, and s-iPTH, the only significant predictors of s-Ca^2+ ^were ethnic background (mean difference of 0.02 (95% CI 0.01, 0.03) mmol/l for Pakistanis vs. Norwegians, p < 0.001) and s-iPTH (mean decrease of 0.03 (95% CI 0.02, 0.04) mmol/l per 10 pmol/l increase in iPTH, p < 0.001). If we excluded s-iPTH from the model, the vitamin D metabolites did still not predict s-Ca^2+ ^levels. S-iPTH seemed to predict s-Ca^2+ ^more strongly in Pakistanis, as Pakistanis had a mean decrease of 0.04 (95% CI 0.02, 0.06) mmol/l per 10 pmol/l increase in iPTH, p < 0.001, whereas Norwegians had a mean decrease of 0.02 (95% CI 0.01, 0.04) mmol/l per 10 pmol/l increase in s-iPTH, p = 0.007. However, the interaction term for ethnic background and s-iPTH on s-Ca^2+ ^was not statistically significant (p = 0.068, adjusted for age and gender).

In a subsample, pH in venous serum samples was 7.54 (95% CI 7.53, 7.54) in Norwegians (n = 429) and 7.51 (95% CI 7.51, 7.52) in Pakistanis (n = 124), p < 0.001.

## Discussion

We found that Pakistanis living in Oslo had low s-1,25(OH)_2_D accompanying their low s-25(OH)D, which may imply a substrate-limited production of 1,25(OH)_2_D. Thus, Pakistanis, who have a 4–5-fold higher prevalence of vitamin D deficiency and secondary hyperparathyroidism than ethnic Norwegians, do not seem to compensate for their vitamin D deficiency by increasing their production of 1,25(OH)_2_D. Interestingly, we found higher s-Ca^2+ ^in Pakistanis compared with Norwegians. A limitation of the study was non-fasting blood samples, which could influence s-iPTH and s-Ca^2+ ^levels. However, adjustment for time since last meal did not alter the observed levels of these metabolites.

It has not been established how the endocrine system handles subclinical vitamin D deficiency, and to which degree this may vary according to ethnic background, age, or body composition. Some studies have observed a compensatory increased production of 1,25(OH)_2_D following reduced s-25(OH)D. Thus Vieth et al. [15] observed an inverse relationship between s-25(OH)D and s-1,25(OH)_2_D in elderly. Several other studies have reported the same inverse association in African-Americans [9,10] and in Asian Indians [1]. However, a usual finding when studying vitamin D deficient subjects of the elderly Caucasian population is a positive relationship between s-25(OH)D and 1,25(OH)_2_D, implying that when s-25(OH)D is low, production of the active hormone may be restricted by lack of substrate [16]. This seemed to be the situation also in our population of adult Pakistanis and Norwegians. The association seemed to be stronger in Pakistanis, who had a higher prevalence of vitamin D deficiency. It is possible that a slight reduction in vitamin D stores may increase compensatory production of 1,25(OH)_2_D at higher concentrations of s-25(OH)D, but that this does not happen when levels are very low such as in our sample of Pakistanis.

It is not clear whether possible alterations in the vitamin D endocrine system may be due to genetic/biologic or lifestyle factors, or a combination of these. Obesity may be a contributing factor, as an inverse association between s-25(OH)D and BMI [17-20] or percentage body fat [21] has been reported. A US study on 302 healthy subjects found lower s-1,25(OH)_2_D in obese compared with non-obese men and women, although the obese subjects had lower s-25(OH)D and higher s-iPTH [22]. The Pakistanis living in Oslo have a high prevalence of obesity as well as a high waist hip ratio for their BMI compared with other ethnic groups [23]. Thus, ethnic differences in the vitamin D endocrine system could, at least partly, be attributable to differences in body size. When studying subjects of Asian background living in Oslo, we observed an increased prevalence of vitamin D deficiency in overweight compared with normal-weight women of Pakistani and other Asian background, but not in men [24].

We are not aware of any published data which have shown that s-1,25(OH)_2_D is inversely associated with BMI in subjects from the Indian subcontinent. In this study, we observed an inverse association between BMI and s-1,25(OH)_2_D in Norwegians but not in Pakistanis. This was, however, apparent only when comparing obese subjects (BMI > = 30) to non-obese (not shown). Differences in BMI or waist hip ratio could not explain the ethnic differences we found in s-1,25(OH)_2_D.

The Pakistanis had low s-25(OH)D, relatively lower s-1,25(OH)_2_D, and high s-iPTH, but they had normal s-Ca^2+ ^when expressed as adjusted to pH 7.40. Although s-Ca^2+ ^is strictly hormonally regulated within a narrow range, the levels were statistically significantly higher in the Pakistanis than in the Norwegians. The results may reflect true higher s-Ca^2+ ^in Pakistanis, or a different pH in Norwegians and Pakistanis. If true s-Ca^2+ ^were similar, our results may arise from a higher pH in the Pakistanis, leading to overestimation of s-Ca^2+ ^values when adjusting the results to a lower pH of 7.40. A more alkaline environment would imply a protection against bone resorption in the Pakistanis. Vaitkevicius et al. found similar plasma pH in African-Americans and Caucasian Americans but lower urinary acid and calcium excretion in African-Americans, and they suggested that alkali administration may be equally important as calcium administration for bone mineralization [25]. Several unidentified factors may contribute to a differing pH and calcium metabolism between ethnic groups. Systemic acidosis is produced by a variety of factors such as menopause, heavy exercise, or dietary acid load, and it is known to induce long-term activation of osteoclasts, in order to release calcium and phosphate into the circulation as a buffer [26]. However, this is not in accordance with our findings on pH in venous serum samples, which suggest a slightly *lower *pH in Pakistanis. If the observed levels in the serum samples reflect true pH in serum, this would imply that the true ethnic difference in s-Ca^2+ ^is even higher than that observed. A true ethnic difference in blood pH has not been established, and would require more sophisticated methods such as measurement of arterial blood gases.

S-Ca^2+ ^was not associated with serum levels of vitamin D metabolites in our study, but s-Ca^2+ ^was strongly inversely associated with s-iPTH in both ethnic groups, suggesting that secondary hyperparathyroidism is induced by low s-Ca^2+^, regardless of ethnic background. However, at any s-iPTH level, Pakistanis had higher s-Ca^2+^. This may suggest a higher set point of calcium in Pakistanis, involving a higher PTH secretion as a response to s-Ca^2+ ^[27]. The differences may also be due to lifestyle factors – such as the dietary acid load, or a combination of several factors. The implications of a higher s-Ca^2+ ^for bone metabolism and bone health in Pakistani immigrants need to be further studied.

## Conclusion

Immigrants with Pakistani background living in Oslo, who had low s-25(OH)D and high s-iPTH, also had significantly lower s-1,25(OH)_2_D compared with ethnic Norwegians. Although obesity was inversely related to s-1,25(OH)_2_D in Norwegians, the lower s-1,25(OH)_2_D in Pakistanis could not be explained by BMI. Interestingly, s-Ca^2+ ^was higher in Pakistanis than in ethnic Norwegians for any s-iPTH in the normal range, also when controlling for age. The explanation for this is unclear given current knowledge of calcium metabolism.

## List of abbreviations used

25(OH)D – 25-hydroxyvitamin D

1,25(OH)_2_D – 1,25-dihydroxyvitamin D

PTH – parathyroid hormone

Ca^2+ ^– ionized calcium

BMI – body mass index

## Competing interests

The author(s) declare that they have no competing interests.

## Authors' contributions

KH performed the data analysis and prepared the manuscript with HEM. AJS and HEM were responsible for collection of the data and organizing of the osteoporosis substudy in the Oslo Health Study. EH was responsible for the blood sample analyses. JAF contributed to the design of the paper. All coauthors have critically revised and approved the manuscript.

## Pre-publication history

The pre-publication history for this paper can be accessed here:


